# Schooling and Identity: A Qualitative Analysis of Self-Portrait Drawings of Young Indigenous People from Chiapas, Mexico

**DOI:** 10.3389/fpsyg.2016.02083

**Published:** 2017-01-10

**Authors:** Moises Esteban-Guitart, Pilar Monreal-Bosch, Santiago Perera, José Bastiani

**Affiliations:** ^1^Department of Psychology, University of GironaGirona, Spain; ^2^Division of Social Sciences, Intercultural University of ChiapasSan Cristóbal de las Casas, Mexico

**Keywords:** literacy, formal education, identity, self-portrait, social change

## Abstract

One of the features associated with schooling and formal education is their decontextualized nature, a characteristic that has been related to the advancement of logical abstract thinking. The aim of this study was to compare and contrast self-portraits through the graphical representations and verbal explanations made by young indigenous people from the Altos de Chiapas with different educational levels ranging from primary school to university. Participants were of the same age. The results show the abstract nature (as opposed to the concrete character) of some of the self-portraits made by the group of university students and the prevalence of individual aspects (rather than social contextual factors) especially within this same group.

## Introduction

The aim of this study was to analyze identity, understood as the definition of oneself, through drawings and the explanations of them given by two groups of indigenous young people with different educational levels from Altos de Chiapas in Mexico.

In a recent study, Esteban-Guitart et al. (2015) found statistically significant differences between the number of personal and social self-concept categories of indigenous youngsters in relation to their sociodemographic situation and educational level. More specifically, personal categories predominated among the young, indigenous university students in comparison to indigenous youngsters with a lower level of formal education (primary school), according to the personal and social self-concept task (PSSC). This methodological technique involves giving each participant a set of cards with the names of various possible identities. These include a range of personal identity terms (such as generous or applied) and a range of social terms (e.g., religion or ethnolinguistic group). The participants were simply asked to choose and rank 5 cards that describe themselves. Of the 18 cards available, 9 were “personal” and 9 were “social.”

The “personal” identity or independence terms refer to psychological traits people use to define themselves (individual orientation or identification, or individual construal of self). The “social” identity or interdependence terms refer to the portion of an individual's self-concept derived from perceived membership in relevant social groups (reference group orientation or identification, or interpersonal construal of self) (Markus and Kitayama, 1991, 2010).

Another recent study contrasted the self-concept among young indigenous people in Chiapas (Mexico). They were divided into three groups: young indigenous rural people with little formal education (rural-traditional); young indigenous students with rural origins (rural-urban); and young indigenous university students with urban origins (urban). The results revealed significant differences between the three groups. The personal categories are given a higher value in the “urban” group while the social categories score higher in the “rural-traditional” group (Esteban-Guitart et al., 2017).

Taking these studies into consideration, along with the existing literature introduced in the next subsection, we expected elements linked to individualism and personal identity to be more prevalent, and descriptions and representations more abstract in the self-portrait drawings and explanations of indigenous university students than in those of indigenous youngsters who had only finished primary school.

## The effects of schooling on identity

The fundamental thesis of Vygotsky and Luria's widely known cultural-historical theory was the material contingency of higher psychological processes. In other words, higher psychological processes like intentional memory, verbal thinking, and identity are the product and result of cultural mediation and of the appropriation of cultural artifacts (Vygotsky and Luria, 1993). In the words of Ratner (2006, p. 13) “we are the products of the products we produce,” meaning that psychological functions like thinking and identity are the outcomes of participating in specific cultural practices through which people appropriate, or learn to use, the repertory of psychological and cultural tools that allow them to increase and regulate their behavior (Rogoff, 2003; Bruner, 2012).

The Bolshevik Revolution of 1917 brought about an “extraordinarily deep and rapid restructuring of historical forms” (Luria, 1971, p. 265) and led to evaluation of Vygotsky, his cultural-historical theory, and his school of thought. At the beginning of the1930s, Alexander Romanovich Luria and other Russian psychologists undertook two expeditions to the Steppes of Central Asia to determine the psychological impact of the 1917 socialist revolution on an ancient, Islamic culture of traditional cotton growers (Luria, 1976). The results obtained from studying Uzbeks and ethnic Kirghiz lent Vygotsky's theory empirical support: cognitive processes like thinking, perception, identity, and problem solving varied according to life circumstances (Nell, 1999).

The data collected by Uzbeks and ethnic Kirghiz suggested that higher psychological functions varied according to the different ways of life and specific realities of the different social groups studied. In other words, the transformation of the economic bases, the eradication of illiteracy, and the changes occurring with regard to religion made possible a revolution in cognitive attitude that Luria (1976) called the shift from practical ways of thinking (intuitive-active) to abstract thinking.

Expanding on the work by Luria, we focus on identity. Specifically, we examine if a similar shift can be found in people's self-consciousness. Indeed, in the study conducted in Central Asia, some interesting differences were found between illiterate and literate participants. The researchers asked how participants assessed their own character, in what way they were different from other people, and what positive and negative traits they could pinpoint about themselves. Illiterate participants from geographically remote areas focused their answers on the concrete, material facts of their lives (‘I’m very well, I've got everything I need but other people haven't, I notice that straight away. Me? I've only got one dress and two housecoats, these are the only things I haven't got enough of', “I want to be OK but now I'm not, I've only got a few clothes, you can't walk around like this in a place you don't know” or ‘I’m kind-hearted, I even talk politely to a little child, and I talk to him courteously'). The participants who had been to school expressed their answers quite differently: “If you ask me how I would describe my character, I could say what my good points are, but you'd best ask my friends what my negative ones are” or “I can't talk convincingly, I've got a weak character, I can't treat people badly and I think that's good” (Luria, 1976, pp.144–160).

Despite the criticisms of the theory and methodology of this study, for example, the low ecological validity of the tasks carried out (Cole, 1988, 2012), much cross-cultural research conducted after Luria's expeditions has examined the effects of schooling and other sociocultural changes on cognition and the development of thinking (Cole and Scribner, 1974; Rogoff, 1981; Scribner and Cole, 1981; Cubero and Ramírez, 2005; Levine et al., 2012).

One of the aspects highlighted in the existing literature along Luria's line of research is the effect that schooling has on the development of abstract, logical-formal thinking. Any formal educational experience, as opposed to an informal educational one, is characterized by its *decontextualization*, by the fact that the learning experience takes place in a setting, in an institution that is culturally constructed for this purpose and is separate from other everyday activity contexts. The learning that is developed is based on representing a reality, on making present and working on, discussing and analyzing a cultural product or content that has been symbolically manipulated: for example, studying the history of Rome without ever having been to Rome. This practice, which is mediated by the symbolic possibilities of language and other semiotic resource systems in use, foments the use of representation and abstraction and the hypothetic-deductive process that defines the type of thinking required for most formal interactions (Rogoff, 1981; Scribner and Cole, 1981).

In the mid-1960s, Greenfield carried out a series of studies in Senegal to analyze the effect of schooling on the development of categorization and conservation. The results showed that Wolof children who were not schooled seemed not to differentiate between believing and thinking and the content of said belief and thought: “It would seem that the unschooled Wolof children lack Western self-consciousness: they do not distinguish between their own thought or statement about something and the thing itself. Thought and the object of thought seem to be one” (Greenfield and Bruner, 1966, p. 92).

According to these studies, schooling seemed to promote self-consciousness in Wolof children, in the sense that they could distinguish between mental acts (believing, thinking) and the external event. Furthermore, schooling increased their ability to categorize or, in other words, to see and deal with a certain stimulus according to different criteria and points of view or use a cultural category (arbitrary and conventional) to manipulate, group and establish relationships between different objects. The example of the study on perception described above usefully illustrates how people who are schooled acquire categories like shape and color and how they establish relationships between elements based on these categories. Greenfield and Bruner (1966, p. 94) concluded that “School seems to promote the self-consciousness born of a distinction between psychological processes and external physical phenomena.”

A recent study analyzed the relationship between formal schooling (understood as a specific cultural practice), autobiographical memory, and the self. The results showed that a certain amount of formal educational experience (becoming literate, basic and university) foments ways of remembering and describing oneself which are very similar to those that characterize the so-called autonomous cultures: the individual as the protagonist, a focus on autonomy and self-definition based on attributes and mental states (Santamaría et al., 2012).

Supported by the work of Olson (1994), de la Mata and Santamaría (2010) sustained that school, as a cultural practice associated with writing, reinforces the modern notion of the individual, conceived as an autonomous entity defined by a set of attributes and mental states.

Despite the problem of making value judgments in which one form of identity and thinking is better than another (Cole, 1988), formal education has historically been the product of processes external to, and generally not invited by, indigenous communities, such as colonization/imperialism, globalization and capitalism-consumerism. It seems that in modern, global, capitalist societies, individualistic, personal identity and abstract thinking are more adaptive (Esteban-Guitart, 2011; Esteban-Guitart and Ratner, 2011). On the other hand, in collectivistic environments, social identity and practical thinking are more appropriate.

In other words, identity conflicts could exist in people who are between rural-traditional and urban-modern environments. If young university students with rural origins return to their communities, the risk of feeling disconnected from more social ways to define oneself is greater due to the prevalence of other models of self and thinking. Indeed, traditional rural communities in Chiapas seem to have progressively adopted values associated with globalization and modern capitalism.

In that regard, longitudinal studies conducted by Greenfield and colleagues analyzed the psychological impact of historical and sociocultural changes on the indigenous people of Altos de Chiapas, in Mexico (Greenfield et al., 2003, 2009; Maynard and Greenfield, 2008; Manago and Greenfield, 2011).

The first observations and analyses date back to1969–1970 and were repeated between 1991 and 1993 and 1997 and 2007. The fact that the study was longitudinal meant that the changes that occurred in the community—the shift from an economy based on subsistence and agriculture to the incorporation of money and commerce and the rise in schooling and urbanization—could be described. These ecological transitions are related to an increase in independent cultural learning and more abstract ways of thinking and representation (Greenfield et al., 2003).

The author and collaborators returned to this community and continued their research between 1997 and 2007. In one of their studies, they analyzed cultural practices and family values ethnographically. More dependence on technology, a rise in individualistic values (autonomy, individual choice), specialization in economic tasks, and more widespread schooling, especially among women, were documented (Greenfield et al., 2009).

Greenfield's theory of *social change and human development* shows how changing sociodemographic ecologies alter cultural values and learning environments and, consequently, the course of psychological development. A worldwide trend toward urbanization, commerce, schooling and technology is taking hold in our environments. Through adaptive processes, these ecological niches encourage and demand individualistic values, fomenting a psychological development pattern of a personal nature (autonomy as opposed to interdependence) and abstract thinking processes (Greenfield, 2009, 2016).

Using drawings as a methodological technique, Keller and colleagues have studied the cultural similarities and differences of preschool-aged children's self-perception. The research was guided by the assumption that specific drawing characteristics would vary cross-culturally according to differences in cultural models and the associated understanding of self and others. In particular, these cultural models included (1) psychological autonomy (characteristic of Western, urban, middle-class contexts), (2) hierarchical relatedness (representative of non-Western, rural, traditional contexts), and (3) mixed cultural models of autonomous relatedness (e.g., non-Western, urban, middle-class contexts; migration contexts). Overall, the studies revealed that figure size, facial depiction, and gender-specific characteristics could be linked to the culturally shaped understanding of self and others in the respective cultural context (Gernhardt et al., 2014, 2015). Regarding the size of the figures drawn, for instance, rural Cameroonian children (who represent the cultural model of hierarchical relatedness, interdependent self-conception) draw themselves alone in a family picture smaller than urban German children (examples of the cultural model of psychological autonomy with an independent self-conception) (Rübeling et al., 2011). However, this line of research did not address, among others, cultural differences due to educational background in drawings made by young people. This is the focus of the research reported here.

## Methods

### Participants

Seventy-two indigenous youngsters ranging in age from 19 to 29 years (average 21.67 years) took part in the study. Of the sample group, 59.7% were women (43 participants) and 40.3% were men (29 participants). Tseltal was the native language of 39 participants (54.2%), Tsotsil of 30 participants (41.7%), Ch'ol of 2 participants (2.8%) and Zoque of 1 participant (1.4%). All of the participants were bilingual: they spoke their indigenous language and Spanish. Slightly more than half (52.8%) were university students (38) and slightly less than half (47.2%) had a primary level of education (34). The participants came from San Cristóbal de las Casas and the surrounding area in a region known as the Altos de Chiapas: Amatenango del Valle, San Juan Cancuc, Pantelhó, Chanal, Chenalhó, Zinacantan, San Juan Chamula, Oxchuc, Huixtán, Tenejapa, San Andrés Larráinzar, and Teopisca. It is an economic and cultural region characterized by enormous religious, political and ethnolinguistic diversity and by the fact that most of its population is rural indigenous people who make their living mainly from agriculture, livestock farming, commerce, and tourism. The region extends over an area of 3717.08 km^2^ and the two predominant ethnolinguistic groups are the Tsotsils and the Tseltals. San Cristóbal de las Casas is the capital of the region, one of the areas of Mexico with the highest indexes of emigration, social exclusion, and poverty (Fábregas, 2006).

The indigenous university students were recruited from the Intercultural University of Chiapas, a higher education institution created in San Cristóbal de las Casas in 2004. The university offers training in six major areas: tourism, intercultural communication, language and culture, sustainable development, law, and medicine. The participants were students from the language and culture degree program. The school has a student body of more than 1200, of which 60 percent are women, and around 50 percent are indigenous. All students were bilingual speakers of one of the indigenous languages (mostly Tzotzil or Tseltal) and Spanish. The university was developed with, but not exclusively for, indigenous groups. The curriculum incorporates the knowledge and languages of the indigenous peoples of Mexico together with the vision, knowledge, and languages of Western culture.

Although the Intercultural University of Chiapas did not require any ethics approval, all participants gave written informed consent to participate in the study.

### Instrument

The self-portrait drawing technique used by Bagnoli (2009) to study the narrative construction of identity in migrant people was employed to elicit participants' representations of themselves. The instructions used were, “Can you try to draw who you think you are at this moment in your life. If you want you can add the people, things, organizations, and activities that are most important or significant to you^1^.” Then the participants were asked to explain what they had drawn. This methodological technique was used because all of the participants had at least a primary level of education and drawing was a familiar activity for them, and also because it is a strategy that has been used with people of all ages and social backgrounds given that it allows for expression through a graphical representation as well as a written explanation (Esteban-Guitart and Vila, 2010; Gifre et al., 2011; Esteban-Guitart, 2012).

While visual methods are not new to the social sciences, it is true that over the last few decades there has been renewed interest in developing and using what the literature calls “sensory research methods” (Mason and Davies, 2009) or “arts based research” (Finley, 2008). These strategies are especially suited to people with comprehension and language production difficulties, for example children in the process of language acquisition, old people, foreigners who are learning the language of the host country, and even people with intellectual disabilities. The use of photographs, drawings, maps, and graphics produced by the participants themselves offers a register or type of insight that is different from that provided by oral or written texts because it allows for more concise representations of key elements of participants' experience (Mitchell, 2011).

Bagnoli (2009) points out some advantages and some drawbacks of the self-portrait technique used in this study. The advantages or positive aspects are that: (a) it encourages reflectiveness in participants, (b) it can facilitate dialogue with the researcher (acting as an “ice breaker”) and, finally, (c) it has potentially enormous evocative power as an image, facilitating and even leading the explanation by means of the oral and written word. However, the author also points out some possible drawbacks associated with the self-portrait drawings. Some participants can feel uncomfortable with the personal and open nature of the technique. (As a representation of oneself, it implies revealing aspects that are associated with privacy). Furthermore, analyzing an image requires a system of categories because it can be an enormously complex and difficult task. Finally, when focusing on one/some participants, the comparison between different cases can be difficult due to the open and personal-private nature of the technique.

Despite the limitations highlighted by Bagnoli, it is considered to be a useful methodological resource within the context of this study and for our research purposes and aims.

### Procedure and data analysis

The Intercultural University of Chiapas in San Cristóbal de las Casas in Mexico was contacted with the aim of authorizing the application of the technique of drawing self-portraits on 40 indigenous university students and 40 indigenous youngsters in the community around San Cristóbal de las Casas who had not been to university but did finish primary school. The criteria were that they should all be able to speak and write Spanish as well as an indigenous language. In the end, of the 80 identity drawings, 3 from the group of university students and 5 from the group of indigenous youngsters with a primary school education were eliminated from the study because the information was incomplete or the task was not carried out properly. The final number of participants was 72: 37 young indigenous university students and 35 indigenous youngsters with a primary level of education. The self-portrait technique was administered by an indigenous professor (Ch'ol) from the Intercultural University of Chiapas.

The interviews were conducted in Spanish. The average time taken to carry out the task was 15 min per participant. To achieve the purpose of the study, the content of the drawings was analyzed according to three systems of categories: (1) concrete or abstract; (2) referring to individual/personal or contextual/social aspects; and (3) the number of references made to self/others. Regarding numbers 2 and 3, the proposal developed by de la Mata and Santamaría (2010) was taken as a reference and adapted to our purposes. In order to analyze self-narratives (autobiographical narratives), de la Mata and Santamaría (2010) focused on the earliest memory elicited by participants. The categories analyzed were the self in the autobiographical narratives (age of earliest memory, individual versus social content, autonomy orientation, self-others ratio, and description of I). We adapted here the categories individual versus social content and the ratio self and others.

To establish coding reliability, all the responses were coded by two members of the research team: the professor of the Intercultural University of Chiapas and a researcher from the University of Girona. To check reliabilities, 30% of all responses were rated by both coders. Kappa indexes, overall and for each participant separately, ranged from *K* = 0.885 to *K* = 0.957.

“Abstract” was understood to mean if the drawing included conventional signs or symbols. Conventional signs were understood to be the representation of a meaning that is unlike, or is not similar to, what it represents. For example, musical notes and mathematical symbols or letters are conventional signs because they are not similar to the reality in the way that a visual sign such as a traffic warning is. Symbols express a meaning, sense or experience by giving an object a special or determinate significance, for example the symbol of the lion to represent strength. In other words, in the drawing, when a relationship was established between the content and what it represents, for example a person dancing or the members of a family, it was codified as concrete. When the reference or content of the drawing did not correspond visually with what was drawn or represented, it was considered abstract. A drawing that referred to the future, when it was removed from the here and now, from the present, was also considered to be abstract. The instructions accompanying the task asked the participant to draw that moment in their life, the present, so if the participant projected into the future or alluded to the past, it was also coded as abstract.

In relation to the individual/personal and contextual/social categories, when the individual predominated as the central element of the drawing and/or the descriptions focused on likes, skills, qualities, attributes and personal activities, it was codified as individual/personal (‘I’m a housewife' -“soy ama de casa”-, ‘I’m a fighter' -“soy una persona luchona”-, “I like listening to music” -“me gusta escuchar música”-). On the other hand, when contextual or social aspect predominated—references to the community, the natural world or society, such as family or friends—in the drawing and its explanation, it was codified as contextual/social. In this case, the individual did not feature in the drawing or was given less importance than other social and/or contextual elements (“I have drawn my community” -“he dibujado mi comunidad”-, “my family is very important, I have their support” -“mi familia es muy importante, tengo su apoyo”-).

Finally, in relation to the references to self/others, the number of times the participant mentioned ‘I’ or “individual” in their self-portrait drawings was counted. This could appear at different moments in the same definition. The references to others, such as friends, a partner or family, were also counted, as in the following definition: “It is me with my parents because they are the most important people in my life because they have supported me wherever they can” (“Soy yo, con mis padres, porque son las personas más importantes en mi vida, ya que ellos me han apoyado en donde puedan hacer”). In this definition, the pronoun I is mentioned once (I am), and there are three references to the participant's parents: “with my parents (…) they are the most important people in my life (…) they have given me support.” In total, there was one reference to I and three references to others (in this case the family). In a brief self-portrait drawing like “I am a housewife” (“soy ama de casa”), the score would be 1 and 0, as there is one reference to I and no mention of others (see Table 1).

**Table 1:** **System of categories used in the analysis of self-portrait drawings and its explanations**.

Abstract	The presence of conventional signs or symbols in the drawing and/or its explanation.	“These musical notes represent who I am”
Concrete	Correspondence between the content of the drawing and/or its explanation and what it represents.	“I am a girl with glasses”
Individual/personal	Answers referring to personal traits, attitudes, beliefs, or behaviors that were not related to other people.	“I am introvert”
Contextual/social	Answers referring to interdependence, demographic places, or groups with which the participants may participate or belong.	“I am a member of the young Catholic group”
Self/others	The number of times the participant mentioned 'I' or 'individual' and the reference to others.	“I am applied” (self) “my friends are very important to me” (others)


To establish that there were statistically significant differences between the group of indigenous university students and the group of indigenous youngsters with a primary school level of education, and given that the variables were ordinal/categorical (concrete as opposed to abstract and individual/personal as opposed to contextual/social) χ^2^ tests were carried out with the exception of the numerical variable of references to self/others (range 0–3). In this case, an ANOVA test (SPSS software version 15.0) was used to compare the averages. Another analysis was conducted of the references to self/others. In order to contrast groups, the number of self was divided by the number of other references for each group.

## Results

This section is divided into three subsections in accordance with the categories used in the analysis conducted in this study. First, the results regarding the concrete versus abstract are described. Second, the self-portrait drawings are analyzed according to the category individual versus contextual. Finally, the drawings are codified in relation to the references to self/others.

### Concrete vs. abstract

The differences between the two groups were statistically significant [$\chi_{\left(1,\;\text{N}\;=\;72\right)}^{2}$ = 19.912, *p* = 0.001]. All of the self-portraits drawn by the group of indigenous youngsters with a primary school level of education were codified as concrete, while 17 of the 38 drawings (44.7%) made by the group of young indigenous university students were codified as abstract.

**Figure 1:**
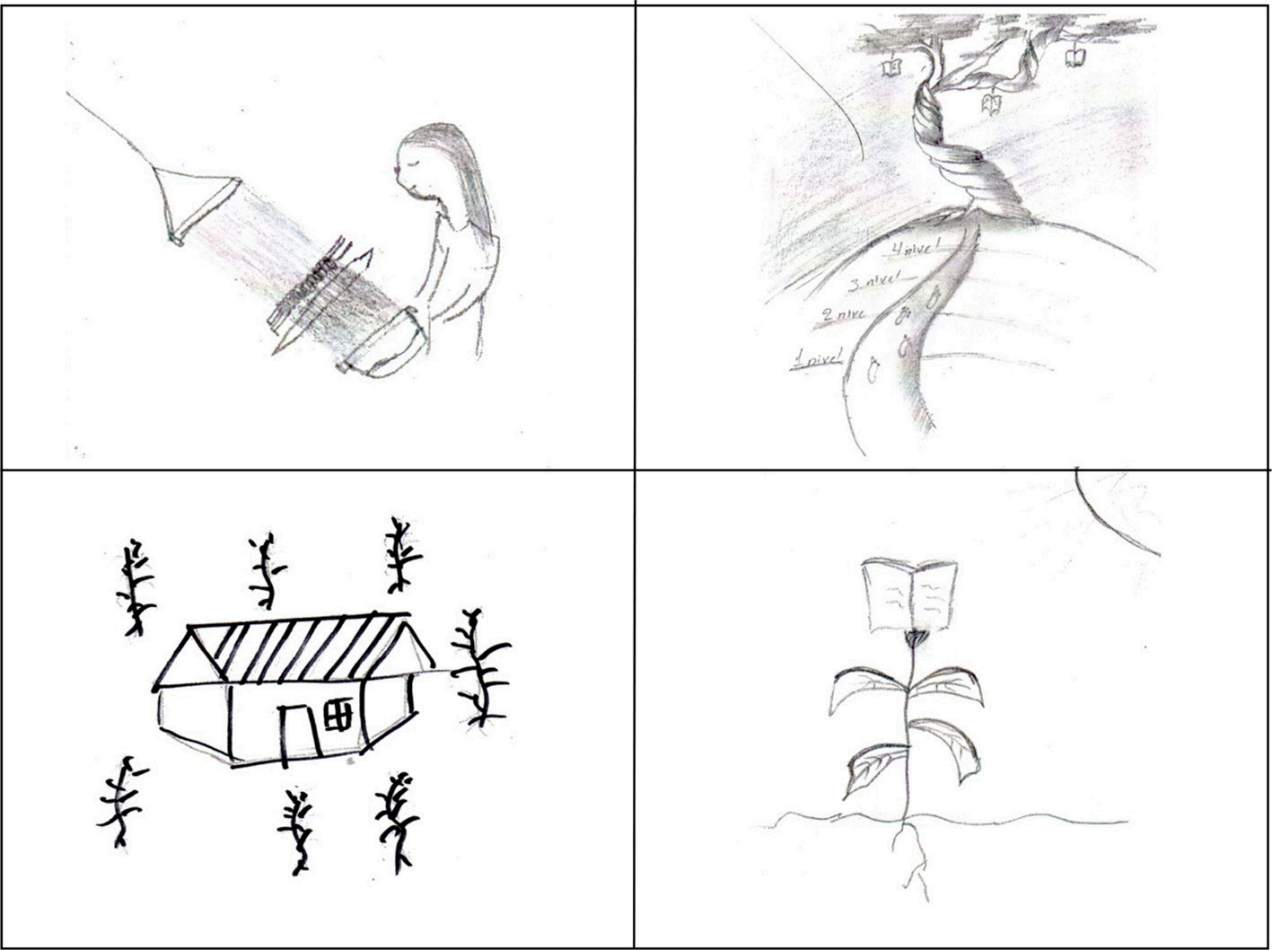
**Examples of “concrete” (on the left) vs. “abstract” self-portrait drawings (on the right) made by participants**.

Figure 1 shows four examples of self-portrait drawings. The drawings that appear in the left-hand column were codified as concrete and were produced by the group of indigenous youngsters with a primary school level of education, while those in the right-hand column were codified as abstract and were produced by indigenous university students.

The top left-hand drawing was made by a 25-year-old woman from Pantelho who speaks Tsotsil and shows herself spinning cloth, which she says “is what I do” (“es lo que me dedico hacer”). Below this there is a picture of a house surrounded by corn drawn by a 21-year-old Tsotsil from Huixtón. “I drew a house where I live. I like my house, it's made of wood and I've got corn around it to eat” (“dibujé una casa donde yo vivo, me gusta mi casa, es de madera y alrededor tengo milpa, para alimentarnos”). Both are examples of the concrete category; in other words, in these drawings there is correspondence with and a relationship between what is being represented, the drawing, and what it represents, the content or meaning. Other examples of concrete drawings describe hobbies and/or daily activities such as studying, dancing or one's occupation (farmer, builder, seller, driver, and housewife).

**Figure 2:**
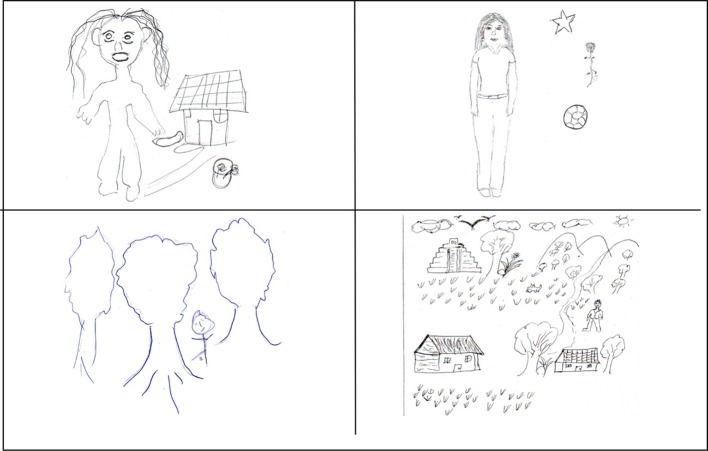
**Examples of “individual/personal” (on the left) and “contextual/social” drawings (on the right) made by participants**.

In the top right-hand corner of Figure 1 is a drawing made by a 19-year-old Totsil from San Juan Chamula who was studying at the Intercultural University of Chiapas. He explained, “a tree of wisdom. We decide how far we want to go” (“un árbol de sabiduria. Nosotros decidimos hasta donde queremos llegar”). Four levels are represented in the drawing with some steps and a path leading to the tree whose leaves are books. The drawing underneath was also made by a 19-year-old from San Juan Chamula and studying at the Intercultural University of Chiapas. It also represents knowledge but, in this case, through a flower. He said, “knowledge where it is born, where I want to learn important things in life, and where great and pacific ideas are born where the seed comes from” (“el conocimiento donde nace el conocimiento, donde quiero aprender cosas importantes en la vida, y donde nace ideas grandes y pacíficas, donde proviene de la semilla”). Despite the fact that both pictures show natural objects, a tree and a flower, the meaning has nothing to do with what is drawn, and in both cases knowledge is symbolized through a book embedded in the plants, in the first case as the leaves of the tree and in the second as the head of the flower. In other examples of drawings codified as abstract, relationships between personality traits and psychological states and life experiences appear, such as bravery and love represented by animals like the lynx and a red flower, respectively. A question mark symbolizing the future and other resources like a star, which for the participant signifies the memory of her mother, also appear (see Figure 2).

### Individual vs. contextual

The content of the drawings and their associated explanations differ according to whether individual/personal or contextual/social elements predominate. Table 2 shows the total number of drawings codified into the two categories related to participants' level of formal education. No statistically significant differences were observed between groups in this sense [$\chi_{\left(1,\;\text{N}\;=\;72\right)}^{2}$ = 1.790, *p* = 0.14]. Therefore, individual/personal aspects predominate over contextual/social aspects in both groups. However, among the young indigenous people at university, the differences between individual/personal and contextual/social references are greater.

**Table 2:** **Individual/personal and contextual/social content according to educational level**.

	**Individual/personal**	**Contextual/Social**	**Total**
Young indigenous people with a primary school level of education	19 (55.8%)	15 (44.2%)	34
Young indigenous people at university	27 (71%)	11 (29%)	38
Total	46	26	72

Figure 2 shows some examples of individual/personal and contextual/social drawings from the two groups. The drawings in the left-hand column were made by participants from the group of indigenous youngsters with a primary school education and the drawings in the right-hand column were made by participants from the group of young indigenous university students. The two examples in which the fundamental or principle content is the individual are at the top and the two examples of contextual drawings are at the bottom.

The top left-hand drawing was made by a 24-year-old from Chanal who defined herself as “a housewife” (“soy ama de casa”). The drawing shows her at home and the emphasis is on her main, daily activity. The top right-hand drawing was made by a 19-year-old Tseltal who was studying at the Intercultural University of Chiapas in San Andrés Larráinzar. The explanation is, “I like drawing and I love playing football and I don't like being hassled. I am a girl but I like Kung-Fu and at night I like seeing the stars shining because when I look at them I miss a person I love so much and my family and my mum who is in heaven” (“me gusta dibujar y además me encanta jugar a futbol y no me gusta que me molesten. Soy una mujer pero me gusta el kung-fu y de noche me gusta ver las estrellas brillando porque al verlas extraño a un ser que yo amo tanto y a mi familia y mi madre que está en el cielo”). In both drawings the protagonist stands out. In the first case, the protagonist is the subject of the definition through the description of an activity that she says she does and, in the second case, through the description of the hobbies and things she likes doing and the reference to her family and her mother who she says she misses.

The drawing at the bottom left was made by a 22-year-old Tsotsil from Huixtan who stated, “I drew trees because I am part of them, we can breathe through trees” (“dibujé los árboles, porque soy parte de ellos, a través de los árboles podemos respirar”). The bottom right-hand drawing was made by a 27-year-old student from the Intercultural University of Chiapas who is from Oxchuc and speaks Tseltal. The participant says, “I represent my community which is close to the natural world and maintains part of its culture, not all of it but a small but very important part, and I identify with that” (“represento a mi comunidad que está cerca de la naturaleza y mantiene parte de su cultura no en su totalidad pero una pequeña parte pero muy importante y me identifico con ello”). In both drawings the context is emphasized as a way of defining oneself.

**Figure 3:**
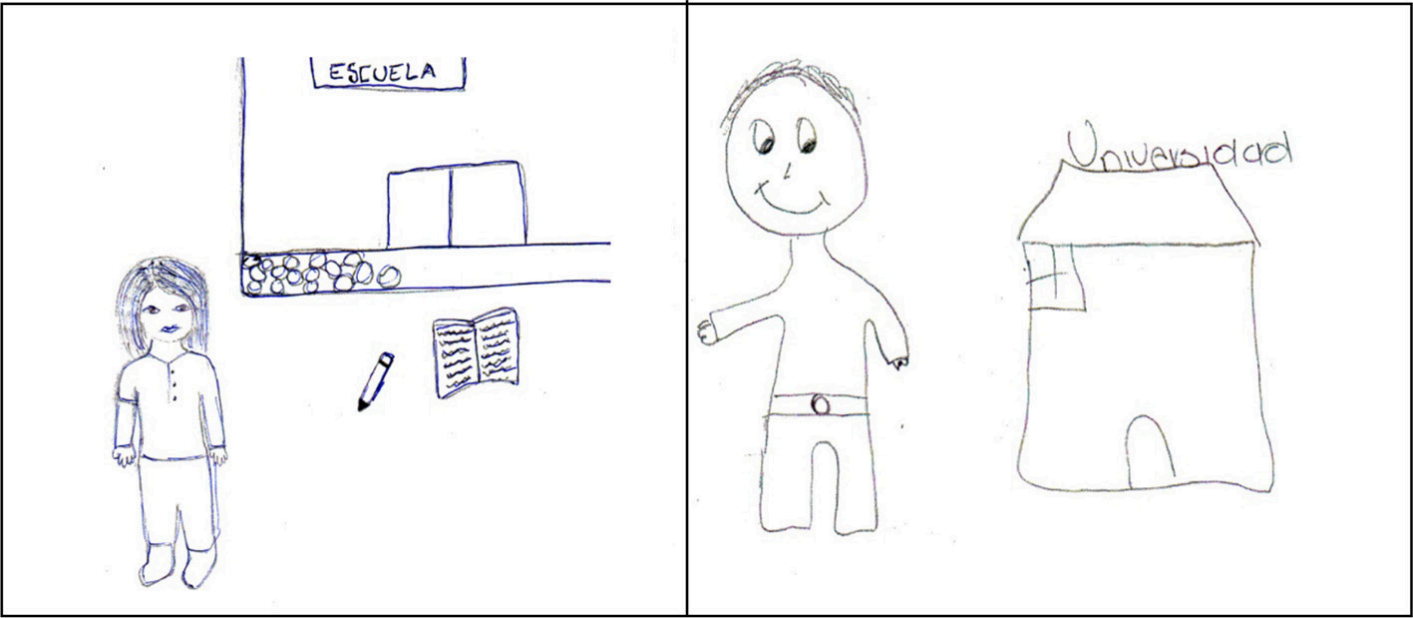
**Examples of two self-portrait drawings made by young indigenous students in which the context of the university appears**.

Among the young indigenous university students, the university itself also appears as a contextual element. It is often referred to as “school” and is linked to their self-definition as students. For example, a 19-year-old Tseltal from San Cristóbal de las Casas who is studying for a degree in Language and Culture at the Intercultural University of Chiapas said, “I am a woman who is in school to prepare to get a secure job” (“soy una mujer que esta en la escuela para poder prepararse para poder tener un trabajo seguro”) (see Figure 3). Next to this image in Figure 3 there is another example, in this case drawn by a 19-year-old Ch'ol from the Intercultural University of Chiapas who said, “I have drawn myself and a university because the most important thing for me is to finish my education” (“me he dibujado yo y una universidad porque lo importante para mi es acabar mis estudios”) (see Figure 3).

In another example a 20 year-old Tsotsil-speaking student from the Intercultural University of Chiapas said, “I am in school studying as a preparation process” (“me encuentro en la escuela como estudiante en un proceso de preparación”). Lastly, in another example, the intention to return to their community after finishing university was expressed. In this case the participant was a 19-year-old Tseltal-speaking young man from Chanal who said, “I am studying in higher education to be of service to the community after I have finished university, to promote my language and to show the organizations in my community how I have studied at different levels of the education system to be a well-prepared professional” (“estoy estudiando en un nivel superior para poder brindar servicio en la comunidad saliendo de la universidad, para fomenter mi lengua y de ir a demostrar en las instituciones de mi comunidad de cómo me prepare durante mis estudios en diferentes niveles de estudios, para ser un profesional preparado”). In this example references were made to two contexts: the young man's community of origin, Chanal, and the university where he is studying.

**Figure 4:**
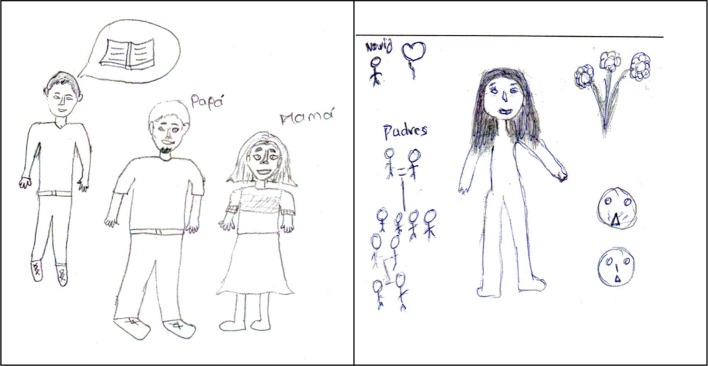
**Examples of two self-portrait drawings coded by the “contextual/social” category**.

An example of a social rather than a contextual drawing is one that makes reference to members of the family. Figure 4 shows the self-portrait drawings of a 21-year-old Tsotsil-speaking university student from San Andrés Larrainzar who said, “It is me with my parents because they are the most important people in my life because they have supported me wherever they can” (“soy yo con mis padres porque son las personas más importantes en mi vida, ya que ellos me han apoyado en donde puedan hacer”). To the right of it is a drawing made by a 21-year-old Tseltal-speaking student who lives in San Cristóbal de las Casas and is studying for a degree in Language and Culture at the Intercultural University of Chiapas. She explained her drawing in the following way: “Because for me the family is very important, knowing that they give me support and especially my boyfriend who also supports me. The flowers are because I love nature and the sad faces are because I feel worried and sad” (“porque para mi es muy importante la familia, saber que te apoya y sobre todo mi novio que he ha dado su apoyo. Las flores porque me gusta mucho la naturaleza, las caritas tristes es que me siento preocupada y triste”).

### Self vs. others

Lastly, small statistically significant differences were found [*F*_(1, 71)_ = 1.914, *p* = 0.040] in relation to the comparison of references to self. Larger statistically significant differences between groups were evidenced in relation to the mention of others [*F*_(1, 71)_ = 7.692, *p* = 0.007]: indigenous young university students make more mention of both self and others in comparison to the indigenous youngsters with a primary education.

As Table 3 shows, in relation to references to self and others (we/they), more references are made to self than to others in both groups (as in the example of the drawing that appears in Figure 3). In this case, the textual corpus, the written explanation written by the indigenous youngsters who drew the pictures, was taken into account. The textual corpus is denser for the group of university students, which explains why there are more references to both self and others in this group (see Table 4).

**Table 3:** **References to self and others**.

	***N***	**Self**		**Others**	
		***M***	***SD***	***M***	***SD***
Young indigenous people with a primary school level of education	34	0.65	0.65	0.29	0.46
Young indigenous people at university	38	0.97	0.67	0.76	0.88

**Table 4:** **References to self and others by the division of references by group**.

	***N***	**Self-references**	**Other references**	**Division**
Young indigenous people with a primary school level of education	34	43	21	22
Young indigenous people at university	38	71	57	14

A complementary analysis was conducted to see the relative predominance of self over other when comparing the two groups. In particular, the number of self was divided by the number of other references for each group (see Table 4).

In summary, the results show that abstract drawings appear in the group of university students, while all of the drawings made by the group of indigenous youngsters with a primary level of education were codified as concrete. Drawings in which no direct visual relationship existed between what was drawn and its content or significance were placed in the abstract category. Metaphors of trees as a representation of knowledge, animals to represent human qualities, the relationship, for example, between the stars and psychological states, and allusions to the future by means of a path or a question mark all appear in these drawings. Concrete activities—often the participant's profession or a description of where they live or work—predominate in the drawings made by indigenous youngsters with a primary school level of education. Content related to the individual predominates in both groups, although the university students' descriptions are more abstract and complex, incorporating mental attributes and states as opposed to just activities. Examples of these include “the sad faces are because I feel worried and sad” (“la cara triste es que me siento triste y preocupada”) (21-year-old Tseltal girl from Tenejapa studying at the Intercultural University of Chiapas), “a girl who fights for what she wants” (“una chica que le gusta luchar por lo que quiere”) (19-year-old Tseltal and university student from San Cristóbal de las Casas), and “I am a playful boy, funny and friendly” (“soy un chavo divertido, amigable y de buena onda”) (19-year-old Tsotsil-speaking university student from Tapachula). As mentioned and illustrated previously, abundant individual/personal content does not mean that there are no references to the community, to the natural world, the university (contextual aspects), or to people, especially the family (social aspects). In the tally of references to self and others in the written texts, both groups make more references to the individual or the self.

## Discussion

The aim of the study was to compare the definitions of self in drawings and their explanations provided by young indigenous people from Chiapas with different educational levels. One group of participants had only finished primary school and the other group was university students.

Previous studies coincide in showing the effects of schooling in developing an autonomous-independent model of self-characterized by emphasizing the individual as a separate, self-contained person focused on internal attributes (personality traits, goals, preferences, and so on) (Maynard and Greenfield, 2008; Greenfield, 2009, 2016; de la Mata and Santamaría, 2010; Santamaría et al., 2012; Esteban-Guitart et al., 2017).

In light of our prior work (Esteban-Guitart et al., 2015, 2017), we expected to find more personal content as opposed to social and/or contextual content in the group of university students. This is the case. However, content associated with individual/personal aspects predominated in both groups.

An important difference between earlier papers is the different method used: the personal and social self-concept task (PSSC) described in the introduction (Esteban-Guitart et al., 2015) and the self-portrait drawings here. In the PSSC, participants were free to select cards, whereas in the current study they had to draw themselves. The instructions allowed them to add people and things that are significant to them but did not allow them to only draw those people and things. In one way, the exercise is guiding them toward a focus on the self, which may explain the strong focus on the self among non-university educated participants.

In general, our results are in line with the mixed cultural models of autonomous relatedness (Kagitçibasi, 1996; Gernhardt et al., 2015) and the trend, identified by Greenfield (2009, 2016), of a global rise in individualism as a result of the transformation of traditional rural contexts characterized by urbanization and the increase in the proportion of people living in urban areas, the rise in schooling, the increase in technology in people's daily lives, and the impact of money, commerce, and tourism. All of these factors have transformed traditional subsistence living for many communities including that of Zinacantan in los Altos de Chiapas (Greenfield, 1999; Greenfield et al., 2003, 2009).

According to Greenfield's *social change and human development* theory (2009, 2016; Manago and Greenfield, 2011), this transformation of the niches and sociodemographic contexts has had repercussions on the settings and practices of teaching and learning which, in parallel, has favored the development of more abstract processes of thinking and reasoning, as well as a trend toward individualism. The study described here illustrates this trend, in particular in the case of indigenous university students who display individual/personal orientation and also abstraction in their drawings and definitions of themselves.

In this regard, Kagitçibasi (1996) disagrees with the traditional distinction between independence-autonomy and interdependence-relation. His concept of the autonomous-relational self-attempts to capture the compatibility of what, according to the author, are human needs: both autonomy and connection or communion. In the family structure, for example, the autonomous-relational self would consist of emotional interdependence and parental authority (Kagitçibasi, 1996). What underlies Kagitçibasi's theory is the reformulation of the concept of autonomy. More specifically, the author distinguishes between two dimensions, agency and interpersonal distance, each of which has two opposing poles. The poles of the agency dimension are autonomy and heteronomy (dependence) and the poles of the interpersonal distance dimension are separateness and relatedness. From here the autonomous-related self lies on a point between autonomy on the agency axis and connection on the interpersonal distance axis.

In other words, our results can be linked by the concept of “the autonomous-relational self” suggested by Kagitçibasi (1996). The participants of the study reported here illustrated the mixed nature of self, incorporating both individual-independent aspects (describing oneself by emphasizing some personality traits for instance) and social or interdependent references (describing oneself by mentioned meaningful social people such as family members). The contextual/social dimension of identity does not necessary imply the inexistence of autonomy, separation-individuation process, or what literature refers to as independent self (Markus and Kitayama, 1991, 2010). In that regard, the autonomous-relational self is suggested to be a healthy synthesis of the agency (independent self) and relatedness (interdependent self) (Kagitçibasi, 2005).

Another explanation of our results refers to the language used to collect data. It is possible that the use of Spanish facilitates the presence of individualistic elements because, in the case of Chiapas (and Mexico to a larger extent), Spanish is the language of schooling, authority, and modernity. Therefore, the use of this language, in contrast to using an indigenous language, in the first place could have primed participants to talk about more modern things, as individualistic ways to express oneself. However, this hypothesis would need to be tested using empirical data.

Another of the elements that we identified after reviewing the existing literature and have incorporated into the analysis of the self-portrait drawings was the distinction between abstract and concrete. To us it is more revealing that representation of self can include abstract versus concrete elements than that people who in general think more abstractly also think abstractly about their identity. In other words, abstract versus concrete are elements of identity defined as “the representation/definition/expression of oneself.”

In this regard, we expected to find more abstract self-portrait drawings among the group of university students. Our results lend empirical support to this hypothesis as the identity drawings of the participants with a primary level of education were codified as concrete, whereas more symbolic and abstract elements appeared in the university students' drawings. For example, they used metaphor and comparison between human qualities (knowledge, love, personality) and elements of the natural world (trees, plants), and their self-definition was based on personality traits and references to the future, which was not the case with the other group. These are drawings that go beyond the description of the here and now and specific daily activities to represent psychological states, aspirations, feelings, expectations, desires, and dreams. This data concurs with the literature review in the introduction, which identifies abstraction as a consequence of participating in decontextualized cultural practices such as school and university (Greenfield and Bruner, 1966; Rogoff, 1981; Scribner and Cole, 1981; Greenfield, 1999, 2009; Greenfield et al., 2003, 2009; Maynard and Greenfield, 2008; Levine et al., 2012; Santamaría et al., 2012).

Finally, we would like to briefly highlight the contributions that we believe the study presented here makes to the existing literature, its limitations and some suggestions for future lines of research.

Our study makes two contributions. First, it adds to existing literature on the consequences and psychological impact of participating in decontextualized formal educational practices. Although the literature concerning cognitive effects and thinking is generally extensive, in relation to our object of study—the identity of young indigenous people from the Altos de Chiapas—it is more limited. Second, we underline the use of the self-portrait projective technique as a mixed methodological resource (visual-graphic and linguistic production) that aims to take the points of view, opinions, and experiences of the participants beyond a conventional and thorough interview (Bagnoli, 2009; Esteban-Guitart, 2012). The self-portrait drawing becomes a testament to the life experiences of the participant that allows us to see the essence located in and distributed throughout human identity (Esteban-Guitart, 2016).

Despite the advantages of using projective techniques such as the self-portrait (i.e., as an image that has potentially enormous evocative power, it encourages reflectiveness in participants) (Bagnoli, 2009), these strategies might not be a culturally relevant practice in particular settings. In order to create culturally relevant methodological tools, we should first analyze how participants present themselves in their local norms and interactional settings. This is an ecological limitation of the methodological strategy used here.

Another limitation of the study refers to the relatively large size of our sample group (72 participants). This fact makes the ethnographic process of describing and understanding the conditions and life contexts of the participants more difficult. It would be interesting to use self-portrait drawings in an ethnographic study of different indigenous young people both with and without formal higher education from the same locality or community. In brief, focusing the study on a relatively small group would allow a more thorough approximation as a result of being able to mix the self-portrait methodological technique with other sources of information: in-depth interviews, personal diaries, and other visual-graphic and narrative-linguistic resources that provide the opportunity to collect data from a multimethodological and autobiographical perspective (Esteban-Guitart, 2012).

To conclude, more studies are needed to document the relationship between educational practices and self-portrait drawings to complement the existing literature about the psychological effects of schooling in a world where literacy and participation in formal contexts is increasingly important.

## Ethics statement

This study was conducted in Chiapas (Mexico). Although the Intercultural University of Chiapas did not require any ethics approval, all participants gave written informed consent to participate in the study in accordance with the Declaration of Helsinki.

## Author contributions

ME: Conception of the work, drafting the article. PM: Data analysis and interpretation, drafting the article. SP: Data analysis and interpretation, drafting the article. JB: Data collection, drafting the article.

## Funding

This work was performed as part of two research projects funded, respectively, by the Spanish Ministry of Science and Innovation (EDU2009-08891) and by La Caixa Foundation (RecerCaixa program, call 2013).

### Conflict of interest statement

The authors declare that the research was conducted in the absence of any commercial or financial relationships that could be construed as a potential conflict of interest.

## Footnotes

^1^The instruction in Spanish was: “¿Podría por favor ser tan amable de dibujar quién piensa que pesonas, cosas, instituciones, actividades que seran más importantes, más significativas, para usted.”
